# The impact of the consumer and neighbourhood food environment on dietary intake and obesity-related outcomes: A systematic review of causal impact studies

**DOI:** 10.1016/j.socscimed.2022.114879

**Published:** 2022-04

**Authors:** Petya Atanasova, Dian Kusuma, Elisa Pineda, Gary Frost, Franco Sassi, Marisa Miraldo

**Affiliations:** a Centre for Health Economics & Policy Innovation, Imperial College Business School, South Kensington Campus, Exhibition Rd, London, SW7 2AZ, UK; b Department of Metabolism, Digestion and Reproduction, Faculty of Medicine, Imperial College London, Faculty Building South Kensington Campus, London, SW7 2AZ, UK; c School of Public Health, Imperial College London, Medical School Building, St Mary's Hospital, Norfolk Place, London, W2 1PG, UK; d Department of Economics and Public Policy, Imperial College Business School, South Kensington Campus, Exhibition Rd, London, SW7 2AZ, UK

**Keywords:** Unhealthy Diet, Obesity, Built food environment, Causal inference methods

## Abstract

**Background:**

The food environment has been found to impact population dietary behaviour. Our study aimed to systematically review the impact of different elements of the food environment on dietary intake and obesity.

**Methods:**

We searched MEDLINE, Embase, PsychInfo, EconLit databases to identify literature that assessed the relationship between the built food environments (intervention) and dietary intake and obesity (outcomes), published between database inception to March 26, 2020. All human studies were eligible except for those on clinical sub-groups. Only studies with causal inference methods were assessed. Studies focusing on the food environment inside homes, workplaces and schools were excluded. A risk of bias assessment was conducted using the CASP appraisal checklist. Findings were summarized using a narrative synthesis approach.

**Findings:**

58 papers were included, 55 of which were conducted in high-income countries. 70% of papers focused on the consumer food environments and found that in-kind/financial incentives, healthy food saliency, and health primes, but not calorie menu labelling significantly improved dietary quality of children and adults, while BMI results were null. 30% of the papers focused on the neighbourhood food environments and found that the number of and distance to unhealthy food outlets increased the likelihood of fast-food consumption and higher BMI for children of any SES; among adults only selected groups were impacted - females, black, and Hispanics living in low and medium density areas. The availability and distance to healthy food outlets significantly improved children's dietary intake and BMI but null results were found for adults.

**Interpretation:**

Evidence suggests certain elements of the consumer and neighbourhood food environments could improve populations dietary intake, while effect on BMI was observed among children and selected adult populations. Underprivileged groups are most likely to experience and impact on BMI. Future research should investigate whether findings translate in other countries.

## Highlights

• Elements of the consumer and neighbourhood food environments could improve populations dietary intake.
• Effects on BMI were observed among children and selected adult populations.
• Underprivileged groups are most likely to experience and impact on BMI.
• Research on the built food environment is limited only to a number of countries.

## 1 Introduction

Cardiovascular diseases and diabetes mellitus are among the leading causes of mortality and morbidity globally (WHO, 2018). One main contributing factor is unhealthy diet, especially one low in fruits, grains, and high in sodium and as shown in the Global Burden of Disease, was responsible for 11 million deaths and 255 million Disease Adjusted Life Years (DALYs) loss in 2017 (Murray et al., 2020). Unhealthy diet also leads to physiological changes associated with obesity and overweight through imbalances between calories consumed and calories expended (Hruby et al., 2016). It is well established that improving nutrition is essential to address the rising obesity rates (Murray et al., 2020; Hruby et al., 2016). Evidence suggests that 83.6% of non-communicable diseases (NCDs) morbidity is caused by exposure to factors in our environment that are amenable to policy interventions, with interventions targeting food environments highlighted as potentially effective in generating population-wide improvements in diets and weight status (Rappaport, 2016).

Food environments have been defined as “the collective physical, economic, policy and socio-cultural surroundings, opportunities and conditions that influence people's food and beverage choices and nutritional status” (Egger and Swinburn, 1997). Within this the human built environment (henceforth the built food environment) has been found to play a key role on dietary quality by shaping the accessibility, availability, and adequacy of food within a geographical area (Gordon-Larsen, 2014). The built food environment has been conceptualized to encompass the *neighbourhood food environment* (defined as the exposure to (measured as availability, density, or distance to) healthy and unhealthy food outlets around places within which individuals gravitate including home, schools, workplaces, and beyond), the *consumer food environment* (defined as attributes experienced by shoppers within food outlets as food types available, price, placement, accessibility, and information). (Glanz et al., 2005; Herforth and Ahmed, 2015).

Previous systematic reviews have provided some evidence of specific elements of the built food environment affecting health and dietary outcomes. For the consumer food environment, previous reviews focused separately on specific in-store interventions related to product placements, monetary incentives, or labelling front-of-pack (FOP) or on-menus, with most reviews focusing only on supermarkets or grocery stores and assessing food intake/purchases (Glanz et al., 2012; Al-Khudairy et al., 2019; Hersey et al., 2013; Bleich et al., 2017; Cameron et al., 2016; An, 2013). Cameron and colleagues indicated that 70% of supermarket interventions report a positive (healthy) effect on dietary intake, but it was not clear which interventions are most effective (Cameron et al., 2016). For the neighbourhood food environment, previous reviews suggested some associations between availability (e.g. count of food outlets near individuals’ homes) and dietary outcomes, and mainly null associations with obesity outcomes (Cobb et al., 2015; Gamba et al., 2015; Feng et al., 2010; Williams et al., 2014; Casey et al., 2014; Caspi et al., 2012; Bivoltsis et al., 2018; Wilkins et al., 2019). Also, the relationships varied across measurements methods, population groups, and food outlet definitions (Cobb et al., 2015; Gamba et al., 2015; Feng et al., 2010; Williams et al., 2014; Casey et al., 2014; Caspi et al., 2012; Bivoltsis et al., 2018; Wilkins et al., 2019).

Notably, previous reviews did not assess the built food environment holistically, with most studies being too narrow. For instance, reviews on the consumer food environment focused on either very specific environments such as the supermarket, or on interventions targeting very specific populations, e.g., medical staff, and importantly not assessing the evidence on the role of exposure to obesogenic food in-stores and its effect on weight (Al-Khudairy et al., 2019). This is a shortcoming as individuals are likely to be affected by the built food environments within which they gravitate through the role played by the availability and affordability of healthy and unhealthy foods (Gordon-Larsen, 2014). Two reviews assessed food environments more holistically by considering broader range of food outlets and/or elements of those outlets (Adam and Jensen, 2017; Mah et al., 2019). However, they still focused on interventional studies inside food outlets disregarding the broader exposure to the built food environment, and focused only on food purchases, thus not assessing how food environments impact obesity outcomes. Also, the reviews on the neighbourhood food environment were limited to the food environment in the context of residential addresses and did not include food environment around schools or workplaces (Caspi et al., 2012; Wilkins et al., 2019).

Secondly, these studies focus on dietary intake and do not assess obesity related outcomes. Given that most literature measures dietary intake through self-reported measures of food intake and/or food purchases that are prone to biases and measurement error (Macdiarmid and Blundell, 1998), it remains important to assess the eventual impact of the built food environment on anthropometric outcomes. Also including obesity outcomes such as body mass index (BMI) might be more reflective of sustained diet changes and long-term weight improvements.

Thirdly, most reviews did not focus on causal impact studies (e.g., randomised control trial, quasi-experimental methods). The lack of causal impact evidence has been well documented in the literature in the last decade (Feng et al., 2010; Wilkins et al., 2019), and is essential for the identification of the effects of exposure to food environments controlling for neighbourhood self-selection bias and competing aspects in the built food environment (e.g., areas with high number of healthy food outlets may also have plenty unhealthy outlets) (Cobb et al., 2015). One review that focused on causal effect (assessing field experiments) but was limited to studies on the impact of subsidies in promoting healthy food consumption drawing on studies with small/convenience samples and short intervention duration (An, 2013).

The aim of this paper is to systematically review and appraise the evidence on the causal impact studies on the relationship between the built food environment (i.e. both consumer and neighbourhood food environments) on both dietary intake and obesity related anthropometric outcomes (e.g. weight, BMI). We therefore build on this literature by considering all studies that assess the causal impact of the built food environment encompassing both the consumer and neighbourhood environments on any outcome of dietary intake and anthropometric outcomes related to obesity.

## 2 Methods

### 2.1 Search strategy and selection criteria

The search strategy of the systematic review was guided by the Population, Intervention, Comparison and Outcome (PICO) method to assess: “What is the causal impact of the built food environment (i.e. consumer and neighbourhood food environments) and interventions targeting the built food environment on both dietary intake and obesity related anthropometric outcomes (e.g. weight, BMI)?“ (Miller and Forrest, 2001). The population focus was on adults and children of any sex, ethnicity, socio-economic status (SES), and country of origin. The outcomes included dietary intake and/or purchases (e.g., intake of fruit and vegetables (FV), sugary drinks, energy-dense foods, fast foods) and obesity related outcomes (e.g., BMI, weight, waist circumference). Including both outcomes (dietary intake and obesity) is important as it can enable the understanding of whether the observed effect of interventions on food consumption translates in improved weight related outcomes. While that is plausible, there is a broad range of factors that may hinder that effect (e.g., short lived interventions, physical activity levels). While assessing the role of these factors is outside of the scope of this review, including both diet and obesity related outcomes enables us to better capture the role they may play in shaping the effectiveness of interventions on obesity outcomes. Generally, the food environment has been conceptualized to include the built food environment– i.e., the consumer food environment (attributes observed inside food outlets) and the neighbourhood food environment (availability, density, or distance to any food outlet around residential addresses, schools, or workplaces) and the organisational food environment (the food available inside homes, schools, or workplaces) (Glanz et al., 2005). We focused on contributions that examine any element of the built food environment including both the consumer and the neighbourhood food environment. We included quantitative studies that deployed methodologies suitable for causal inference. While the gold standard would be randomized controlled experiments, given that randomized controlled experiments are not always feasible in the context of policies and interventions, we also considered quasi experimental methods. The challenge for causal inference in non-randomized experiments is the ability to find an appropriate counterfactual to compare the outcome of interest of treated with. The ideal counterfactual would be what would have happened if treated had not been treated. In non-randomized interventions that is not observable and therefore the counterfactual needs to be estimated in a different way. Therefore, beyond RCTs, we also include papers that deploy statistical techniques that have been developed for that purpose in the context of observational data and are suitable to identify causal effects, namely, differences in differences, instrumental variables, regression discontinuity design, interrupted time series and natural experiments (Varian, 2016). These methodologies enable accounting for endogeneity and selection issues that may lead to biased and spurious estimates, and also encompass most of the elements of the Bradford Hill criteria notably, Experiment, Strength, test of significance (Hill, 1965).

Therefore, we excluded papers focusing on clinical sub-groups, qualitative studies and quantitative studies without clear experimental or quasi-experimental designs. Studies claiming to use quasi-experimental designs but did not address key endogeneity issues were also excluded. Further, we excluded contributions examining the organisational food environment (defined as environments and practices within schools, workplaces and homes) as these have been identified in the literature as separate and complex elements of the food environment where different mechanisms play a role on its effect on diets and obesity (Glanz et al., 2005); we have also not focused on the online food environment as there are different social and psychosocial mechanisms that shape the impact of each type of food environment on behaviours and the demographic characteristics of an online shopper are not representative of the population as a whole (Sacks et al., 2011). However, studies that utilized a 3D virtual shop that simulate a real shopping experience were included. PICO table reported in Appendix 1.

A systematic search was conducted on MEDLINE, Embase, PsychInfo (via Ovid), EconLit (via EBSCO) to identify all evidence on food environment elements influencing dietary intake and obesity published between database inception and March 26, 2020. The search string included terms related to the following: (dietary intake OR obesity) AND (food outlets OR food environments) AND (geolocation, proximity, distance). Also, we conducted a bibliography search from full-text articles meeting the selection criteria to include further articles that were missed by our search strategy. We have not included grey literature given the scope of the review is to focus on quantitative analysis using peer reviewed contributions suitable for causal inference. Full search is provided in Appendix 2. No limits or filters were set on the search. A protocol was not prepared.

### 2.2 Data extraction

Literature search results were exported from the databases into Covidence software. The articles were individually screened by three reviewers by assessing titles, abstracts, and full text articles, based on inclusion and exclusion criteria. Authors voted on whether an article should be included in the review. Conflicts were resolved through discussion between authors and in consultation with two other authors where needed.

From each study fitting in the criteria, we extracted author, year of publication, study design, intervention (type, duration, measurement), objectives, exposures, outcomes, data sources and measurements, population, results (direction of effect and p-values), statistical analyses, limitations (attrition rates, missing data, etc.) and country. When p-values were not provided, they were calculated using traditional formulas (Altman and Bland, 2011).

### 2.3 Quality assessment

A risk of bias and a certainty of the evidence assessment were conducted by the same three reviewers via the CASP protocol for randomized control trials and cohort studies which enabled to systematically assess the trustworthiness, relevance, and results of the papers (--, 2021). Each study was evaluated according to the following quality criteria: selection bias, attrition rates, intervention duration, type of outcome and exposure measurement (objective or subjective), confounding factors, being a field experiment (Harrison and List, 2004), generalizability. The scoring tool consisted of 10 criteria, with scores 1 or 0. A final quality concern score was calculated by summing the points for each study. The evaluation of each study and its quality score is provided in Appendix 3.

### 2.4 Data synthesis

Data extracted from articles fitting the inclusion criteria were analysed using narrative synthesis approach in systematic reviews consistent with best practice (Popay et al., 2006). We grouped the included studies based on the food environment setting (consumer or neighbourhood). We created subgroups based on the intervention type (e.g., financial incentives) and exposure to the food environment (e.g., healthy, or unhealthy food outlets according to the North American Industry Classification System as this was the classification predominantly used in the assessed studies) (North American Industry C, 2021). Accordingly, unhealthy food outlets were fast-food restaurants, and convenience stores; healthy outlets were supermarkets, farmers markets, and grocery stores. Dietary intake and obesity outcomes were compared for children and adults separately across their results statistical significance (p-values at the 95% confidence interval) and direction of the effect (i.e., positive, or negative). We then critically appraised these results based on each study score from the criteria in the risk of bias assessment which encompasses, among other characteristics, study design, data sources and measurements, and generalizability. To explore possible causes of heterogeneity among study results we compared and evaluated the studies according to their methods used to draw causal inference, the key intervention components and the subpopulation studied. Paragraphs in the results section are structured to present first findings on dietary intake and then on obesity separately for children and adults. Effectiveness was determined based on vote counting of the number of studies with high quality scores reporting similar results in terms of direction and statistical significance. Studies deploying more robust methods for causal inference were given more weight in the evidence appraisal (--, 2021).

Ideally, a formal meta-analysis should be conducted to provide quantitative estimates of the effect of exposure to the consumer and neighbourhood food environment on diet and obesity. This requires the exposure type and outcome measure across studies to be sufficiently homogeneous. However, among the studies included in this review, few adopted the same experiment strategy, exposure setting, and outcomes also substantially differed. Therefore, the dissimilar nature of the studies included precludes meta-analysis. This study was thus limited to a narrative synthesis of the included studies with general themes summarized.

## 3 Results

### 3.1 Data extraction and quality assessment

**Fig. 1:**
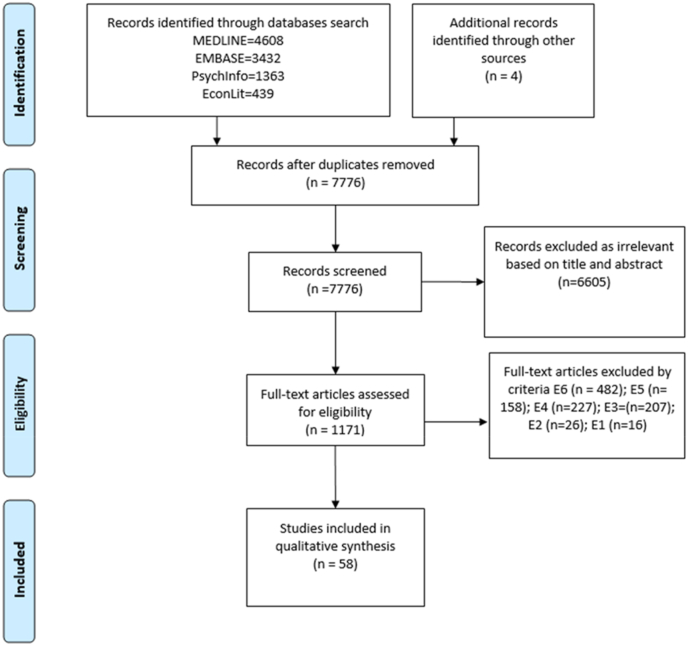
PRISMA flowchart of the study selection process.

Articles were assessed based on inclusion and exclusion criteria described in Table 1. The search yielded 10 033 studies, 2257 of which were removed as duplicates, leaving 7776 for assessment. Following the study inclusion/exclusion criteria and study selection process 58 papers were included in the narrative synthesis. Fig. 1 represents a PRISMA flowchart of the study selection process (Page et al., 2021). 47 studies were low risk of bias, while only four high risk and seven medium risk. The most common quality concerns related to reliance on self-reported data for dietary intake and BMI, not being natural field experiments (if participants were unaware, they were part of a study) (Harrison and List, 2004), and focus on specific populations impacting generalizability. Given our inclusion criteria on research design, other quality concern points were well accounted for as studies randomly selected and allocated participants, attrition rate was low or well accounted for, objectively measured exposure, had long intervention durations, and reported well results (see Table 2) (see Table 1).

**Table 1:** List of inclusion and exclusion criteria.

Inclusion	Exclusion
General populations, any age, any geographic region	E1: Specific clinical groups such as pregnant women, athletes, participants with specific disorders; very specific subgroups (incarcerated, isolated populations)
Studies focusing on dietary outcomes (e.g. FV, SSBs, nutrients intake/purchases) and/or obesity	E2: Studies focusing on disease related outcomes (e.g., diabetes, blood pressure, HIV)
Studies examining the effect of the built food environment on dietary intake and/or obesity	E3: Studies that do not make associations between at least one built food environment metric (intervention) and dietary intake and/or obesity (health outcome)
Quantitative studies	E4: Not quantitative studies, i.e. qualitative studies, policy analysis, socio-economic studies, systematic reviews, meta-analysis, protocols, proposals, etc.
Studies focusing on the consumer and/or neighbourhood food environment, including studies simulating one of those (e.g. 3D stores)	E5: Studies not focusing on the neighbourhood or consumer food environment. Studies focusing on the organisational food environment and on online food environments.
Causal inference methodology	E6: Studies not using causal inference methodology

**Table 2:** Overview of selected studies.

Author/Year/Country	Design	Participants	Intervention	Intervention Duration	Outcome	Outcome Measure	Intervention Measure	Environment Element	Results
Consumer Food Environment *In-kind and Financial Incentives*									
Hobin et al. (2012) Canada	RCT	337 children aged 6–12 years	Premium toy offered with healthier meal options	1,5 months	Purchases of meals that meet nutritional criteria^a^	Tracking purchases	Two conditions- healthy vs unhealthy	Fast food restaurant	Positive- purchases of healthy meals when offered with toy (p < 0.01)
Karpyn et al. (2017) USA	RCT	755 children, limited demographics info	Animal cartoon characters paired with FV	1 month	FV purchases	Purchases tracked by redeemed tickets	Paired vs not paired with an animal cartoon characters	Shops in a local Zoo	Positive - purchases of FV when offered with toy (p < 0.01)
Geliebter et al. (2013) USA	RCT	47 adults, general sample	50% discount on FV, water, diet sodas	2 months	FV and SSB intake and purchases + BMI	24 h dietary recall + measuring body weight, and body composition	Two conditions – 50% discount vs no discount	Two supermarkets	Positive – FV intake (p < 0.05) and purchases (p < 0.05), only FV intake was maintained at follow up; Null – SSB and BMI
Waterlander et al. (2013a) Netherlands	RCT	151 adults; low-income sample	50% discount on FV + education	6 months	FV intake and purchases + BMI	Survey + supermarket register receipts	Four conditions – 1) 50% discount 2) 50% discount + education; 3) education 4) control	Four supermarkets	Discount only- Positive -FV intake and purchases (p < 0.05); Null - BMI
Ball et al. (2015)Australia	RCT	574 women; general sample	20% discount on FV + skill building	3 months	FV and SSB intake and purchases	Survey + supermarket register receipts	Four conditions – 1)20% discount 2)20% discount + skill building, 3) skill building 4)control	Two supermarkets	Discount only- Positive – F intake (p < 0.01) and FV purchases (p < 0.05) Null – SSB intake and purchases
Blakely et al. (2011) New Zealand	RCT	1104 adults; general sample	12.5% discount on 1032 healthy items + nutrition education	6 months	Purchases of healthy products, nutrients^b^	Purchases tracked by instore loyalty card	Four conditions – 1)12.5% discount, 2) 12,5% discount + education; 3) education; 4) control	Any supermarket	Discount only-Positive- increase in healthy products purchases (p < 0.05)
Polacsek et al. (2018) USA	RCT	354 low-income adults	(two for one) 50% discount on FV	4 months	Purchases of FV (spent overall on FV)	Tracked with loyalty card	Two conditions – (two for one) 50% discount or control	1 supermarket	Positive - Increase in FV purchases (p < 0.05)
Harnack et al. (2016) USA	RCT	279 low-income adults	30% discount on FV; restriction on SSB and sweet baked goods	3 months	Dietary intake + BMI	Dietary intake via 24 dietary recall; BMI measured by trained personnel	4 conditions: 1) 30% discount; 2) 30% discount + restriction; 3) restriction; 4 control	Any food store	Incentive and Incentive + Restriction Positive- increased intake of fruit (p = 0.05); improved Healthy Index (p < 0.01); Negative – decreased calories from restricted foods (p < 0.01); Null - BMI
Jilcott Pitts, (2018) USA	DID	537 adults, low income	Opening of a discount supermarket;	2 months	FV, SSB intake + BMI	Surveys	Compared to a similar neighbourhood; store audits to examine shops within 5-mile distance	One supermarket	Null – Dietary Intake, SSB, BMI
Gopalan et al. (2019) South Africa	RCT	2841 adults, normal to higher SES	Up to 25% cash back monthly on healthy food purchases	6 months	Purchases of healthy foods^c^	Purchases tracked via membership card or credit card	Incentive and control group	Grocery store chain	Null – Purchases of healthy foods
Kral et al. (2016) USA	RCT	54 racially/ethnically diverse men and women, aged 40 to 70 living in Philadelphia	1 dollar incentive for each transaction of healthy foods	3 months	Intake and purchases of low fat/low sugar foods + BMI	Purchases tracked with grocery receipts and self-reported 3-day food record; BMI collected by trained personnel	Incentive and control group	Grocery stores	Positive- Vegetable intake (p < 0.05) Null- BMI, other dietary intake metrics
Waterlander et al. (2013a) Netherlands	RCT	109 low-income adults	50%,25%,10% price discount and food label on healthy products	One time experiment	Healthy products purchases	Tracking participants purchases	3-levels of price reduction x 3 types of labels on healthy foods	In a lab using a 3D web-based supermarket	Positive- 50%, 25%, 10% discount on purchases of healthy products (p < 0.01) Null- Labelling on purchases
Guan et al. (2018) USA	DID	2500 households with varied backg	Targeted coupon on healthy or less healthy foods	14 months	Healthy items purchases	Transactions data from retail analytics firm Dunnhumby	Receiving a targeted coupon vs control	5 stores from a single chain retailer	Positive – increase in purchases of healthy items (p < 0.01)
Bernales-Korins et al. (2017) USA	RCT	45 overweight or obese adults, 34 females and 11 males	50% price discount on FV	2 months	FV purchases and intake	Purchases tracked via scan card, dietary intake with 24 h dietary recall	Discount vs control group	4 D'Agostino supermarkets in Manhattan NYC	Positive -Purchases (p < 0.01) and intake of FV (p < 0.05), only intake of FV maintained after discount ended
Smith-Drelich (2016) USA	RCT	130 adult participants, general sample	Up to 50 US dollars cash back after the 3 weeks intervention	3 weeks	Vegetable intake and purchases	Purchases tracked via receipts; Vegetable intake via survey	Cash back payment vs control group	Any vegetable retailer	Positive -vegetable purchases (p < 0.01). Null -vegetable intake
Franckle et al. (2018) USA	RCT	148 low-income adults from an urban Latino community	25$ coupon for in-store purchases if refraining from red labelled beverages	5 months	SSB intake and purchases	Survey + purchases tracked via instore loyalty card	Coupon vs no coupon	Urban supermarket	Negative – decreased consumption (p < 0.01) and intake (p < 0.01) of red labelled SSB products
Banerjee and Nayak (2018) USA	RCT	100 low-income adults	40- or 60-dollars coupon card + education on healthy eating	1 week intervention	Nutrients purchased	Purchases tracked via store cards	5 groups- Coupon + Education; only Coupon; only Education; control	Two local Piggly Wiggly stores	Negative – Coupon + education on unhealthy foods purchases (less calories, sodium, fat (p < 0.01) Null -Coupon only
*Food Saliency and Information*									
Anzman-Frasca et al. (2018)USA	RCT	58 families, with one 4-to-8-year-old child; general population	Placemats featuring two heathy kids' meals	2 months	Healthy meals intake and purchases according to criteria^d^	Survey + plate waste measurements	Two conditions – placemat featuring healthy kid's meals vs no info on placemat	In a quick-service restaurant	Positive- increased consumption (p < 0.05) and purchases (p < 0.05) of healthy meals when placemat featuring healthy meals presented
Cantor et al. (2015) USA	DID	7,699 consumers	Calorie Menu labelling in fast food chain restaurants	5 years	Levels of calories or other nutrients purchased	Itemized cash register receipts and survey responses	Levels of calories or other nutrients purchased	4 Fast-food chains	Null
Vadiveloo et al. (2011) USA	DID	7,699 consumers	Calorie Menu labelling in fast food chain restaurants	.2 years	Favourable, unfavourable food purchasing patterns	Surveys	Beverage, salads, fries, addition of cheese to menu items, desserts purchased. Frequency of fast-food consumption per week	4 Fast-food chains	Null
Knowles et al. (2019) UK	RCT	56 adults; general sample	Placing unhealthy snacks further away and healthy snacks closer	..NA	Snacks intake - 250 g chocolate M&M's or 250 g mixed fruit	Survey + measured consumption of healthy/unhealthy snack	4 conditions - both snack types proximal, Fruit proximal, Chocolate proximal, and both snack types distant,	In an experimental lab	Positive- proximal items were sig. More consumed (p < 0.05)
Marty et al. (2020) UK	RCT	1743 adults, general sample	Calorie menu labelling + availability of low-calorie meals	NA	Calories purchased	Purchases ordered	4 conditions: 1) calorie menu labelling; 2) availability of low-calorie meals; 3) 1 and 2; 4) control	In a simulated virtual fast-food restaurant	Negative – availability of low-calorie meals increased purchases (p < 0.01); Null - labelling
Gittelsohn et al. (2013) Navajo Nation, USA	RCT	145 adults; Navajo tribal members	Promoting healthy food via shelf labelling, exposure to healthy products and information on healthy habits	14 months	BMI	Height and weight measured by trained data collectors but not for all participants	5 Intervention vs 5 control sites	10 store regions^e^	Negative- decreased BMI due to intervention exposure to (p < 0.05)
Milliron et al. (2012) USA	RCT	153 adults; general population	Promoting healthy food via shelf signs, tips, and signage	4 months	Nutritional intake and purchases^f^	Survey + analysing participants shopping basket	Two conditions – receiving explanation and information on promoted healthy foods vs no information	One Supermarket	Positive – more purchases and intake of F (p = 0.01) and V (p = 0,05)
Kristal et al. (1997) USA	RCT	120 adults	50-cent coupon for FV, store signage for FV and nutrition information	8 months	FV intake	Survey	Intervention vs control	8 supermarkets	Null
Petimar et al. (2019a) USA	DID	2971 adults, 2164 adolescents and 447 children Mostly non-white (60–84%) across all samples	Calorie menu labelling	1 year	Calorie intake	Survey + receipt checks	Before and after calorie labelling at McDonald's in 2012 compared to a group of control restaurants^g^	37 McDonalds restaurants	Null
Petimar et al. (2019a) USA	ITS	..NA	Calorie menu labelling	3 years data	Calorie purchases	Transactions	Before and after implementing calorie menu labelling	104 chain restaurants	Negative right after implementation (p < 0.05); null after 1 year follow up
Finkelstein et al. (2011) USA	DID	NA	Calorie menu labelling	1 year	Calories purchases	Transactions	Before and after implementing calorie menu labelling and comparing to a no calorie menu labelling county	Fast food restaurants chain (Taco Time)	Null
Papies et al. (2014) The Netherlands	RCT	99 normal or overweight adults, 95 female, 4 male, mainly low educational level	Health prime was flyer of a low-calorie recipe with stated calories and health benefits	5 days	Purchases of energy-dense snack foods	Purchases tracked by receipts	Health prime vs control	A local grocery store	Null for normal weight participant; negative for overweight (p < 0.05)
Hammond et al. (2013) Canada	RCT	635 adults in Canada	Calorie labelling; traffic light labelling of calories, sodium, fat, sugar	5 days	Calorie and nutrient intake and purchases)	Purchases tracked via receipts; trained staff collected and weight leftover food and beverages to estimate nutrient/calories intake	4 groups – 1) calories label only; 2) Calorie traffic light; 3) Multi-traffic light; 4) none	In a lab showing an adjusted Subway menu	Negative -calorie labelling (p < 0.05) Null- traffic light labelling
Gustafson and Prate (2019) USA	RCT	115 low income adults	Tailored front of pack labelling (text and images)	6 days	Healthy products purchase	Self reported	Tailored label vs generic label vs control	At a local grocery store	Positive – tailored and generic label increase healthy food purchases (p < 0.01)
Grummon et al. (2019) USA	RCT	400 adults	Health warning on SSBs policies	1 year	Food purchase	Questionnaire	1 health warning arm and a control arm	Life-sized replica of a convenience store	Negative-health warning arm lower SSB purchase (p < 0.01)
*Availability/Accessibility*									
Gittelsohn et al. (2017) USA	RCT	385 children and 387 caregivers, low income, 90% African American	Increase the stocking and promotion of healthy food products inside store	Wave 1 - 9 months wave 2 - 11 months	Intake of low sugar, low fat products + SSB	Survey	14 intervention neighbourhoods vs 14 control	55 corner stores and 30 carryout	Positive-Children (p < 0.05) Null- Adults
Lent et al. (2014) USA	RCT	767 4th and 6th grade low-income students	Increase supply of healthier products and identify them via shelf signs	6 months	Purchased nutrients + BMI	Bag checks. + trained staff measured weight and height	12 control vs 12 intervention corner stores	24 corner stores near schools	Null – Nutrients, BMI
Trude et al. (2018) USA	RCT	509 low-income youth from African American origin, living 1.5-mile buffer zone from participating store	Increase supply of healthier food and beverage options and adding signs to identify them	Wave 1: 7 months wave 2: 8 months	Healthy food purchases and calorie intake of sugar and SSB	Purchases and intake were self-reported	Intervention neighbourhoods vs control	3 wholesalers, 30–40 corner stores and carryout restaurants	Positive -Healthier food purchases (p < 0.05) Negative -kcal from sweet snacks (p < 0.05) Null - kcal from SSB and FV
Jilcott Pitts (2018) USA	DID	502 low-income adults	Increase the stocking and promotion of healthy food products inside store	6 months	FV intake	Survey + customer bag-checks, Veggie Meter	Validated audit tool to assess availability in-store conditions	16 small food retailers	Null
Cummins et al. (2014) USA	DID	656 low-income adults	Opening a new supermarket	4 years	FV intake + BMI	Telephone survey	Compared to a similar neighbourhood	One supermarket	Null
Dubowitz et al. (2015) USA	DID	1372 adults, low income	Opening a new supermarket with 30% of ﬂoor space to perishable food items and 500 ft2 to fresh produce	3 years	SoFAAS^h^ + FV intake + BMI	Survey + 24 dietary recall	Compared to a similar neighbourhood	Full-service supermarket	Positive-SoFAAS (p < 0.05); Null- FV intake and BMI
Elbel et al. (2015) USA	DID	2172 caregivers low income	Opening a new supermarket with 30% of ﬂoor space to perishable food items and 500 ft2 to fresh produce	1 year	Nutrients' intake	Street-intercept surveys	Compared to a similar neighbourhood	Full-service supermarket	Null
Elbel et al. (2017) USA	DID	3998 adults, low income	Opening a new supermarket with 30% of ﬂoor space to perishable food items and 500 ft2 to fresh produce	1 year	FV intake	Street-intercept surveys	Compared to a similar neighbourhood	Full-service supermarket	Null
Laska et al. (2019) USA	DID	3,039 store costumers	In-store healthy food stocking requirement policies	1 year	Healthy food customer purchase, healthfulness of home food environments	In-person store assessment	Compared to those in a nearby control city	Supermarkets and WIC authorised stores	Null
Neighbourhood Food Environment *Healthy Food Outlets*									
Zeng (2019) USA	DID	293 124 public school children; general population	Supermarket openings and closings	3 years	BMI	BMI collected by trained personnel	Data obtained from ACHI, counting number of supermarkets within a buffer of 1-mile (urban), 5-mile (rural) radius	Supermarket	Positive – supermarket closures increase BMI (p < 0.05) Negative = supermarket openings decrease BMI (p < 0.05)
Jilcott Pitts (2018) USA	DID	537 adults, low income	Distance to the primary food store	1 year	FV Intake	Survey	Data obtained from Reference USA; calculated distances along the street network from each address to the primary store	Supermarket	Negative – the further the distance to primary store the less FV consumed (p < 0.05)
Zhao et al. (2014) USA	RCT	3519 families, low income	Density of food outlets	1 year	BMI	Survey	Density calculated as the ratio of the number of food outlets to the population at the ZIP code level	Fast food restaurants, grocery stores	Null
Leone et al. (2018) USA	RCT	142 adults; low income	Availability of a veggie van	6 months	FV intake and purchases	Survey + purchases	6 intervention sites vs 6 control	Mobile fresh produce market	Null
Olsho et al. (2015) USA	DID	35 606 adults	Availability of a veggie van	6 years	FV intake	Survey	Compared to a control neighbourhood	Mobile fresh produce market	Null
Kapinos et al. (2014) USA	RCT	1935 first year university students living in dormitories	Availability of food outlets within ¼ mile of residency	1 year	BMI	Survey	Number of grocery stores, restaurants (fast-food, sit-down, and coffee shops) within ¼ of a mile	Grocery stores, fast-food and sit-down restaurants, Coffee shops	Grocery stores negative (p < 0.05); Fast-food, sit-down restaurants and coffee shops null
*Unhealthy Food Outlets*									
Alviola et al. (2014) USA	IV	942 obese students in kindergarten, grade 2,4,6,8,10.	Availability and proximity of fast-food restaurants around schools	2 years	BMI	BMI screenings, height and weight measured	Fast food outlet locations from Dun & Bradstreet business lists, GIS measuring 1 mile radius distance to outlets	Fast food restaurants	Positive (p < 0.05)
Asirvatham et al. (2019) USA	IV	First:1 362 306; Second: 2739 students in grades 1-9	Availability and proximity of fast-food restaurants around schools	6 years	BMI	BMI screenings, height and weight measured	Fast food outlet locations from Dun & Bradstreet business lists, GIS measuring 1/3,2/3 and 1 mile distance to outlets	Fast food restaurants	Positive- 1/3 miles (p < 0.01); 2/3 miles (p < 0.01); 1 mile (p < 0.01)
Wang and Shi (2012) China	DID	185 children aged 6–18: general population	Density of food outlets (wet markets; supermarkets; fast food restaurants)	3 years	Nutritional intake^i^	Survey	Count the number of food outlets within 5 km radius	Wet markets, supermarkets, fast food restaurant	Positive- wet markets (p < 0.01). Null - supermarket and fast-food restaurant
Qian et al. (2017) USA	IV	530 628 children	Density of food outlets within a half mile from residential address	6 years	BMI	BMI screenings, height and weight measured	Fast food outlet locations from Dun & Bradstreet business lists,	Fast food restaurants	Positive –fast foods within one and half a mile were positively significant only for girls (p < 0.05)
Chen et al. (2013) USA	IV	3550 adults; general population	Density to fast food outlets	1 year	BMI	Survey	Count fast food restaurants within 0.5-mile buffer of participants address	Fast food outlets	Positive – adults from medium and urban density (p < 0.05)
Dunn et al. (2012) USA	IV	1019 adults; general population	Availability of fast-food outlets	1 years	Fast-food intake + BMI	Survey	Number of fast foods within 1 mile	Fast food restaurants	Positive- black and Hispanic participants – 1 mile (p < 0.05); 3 miles (p < 0.01)
Dunn et al. (2010) USA	IV	146 954 adults; general population	Availability of fast-food outlets	3 years	BMI	Survey	Count fast food outlets	Fast food restaurants	Positive- black and Hispanic participants (p < 0.05)
Rummo et al. (2017) USA	IV	12 174 individual adults' data	Availability of food outlets	25 years	BMI	Measured by trained staff	Geocoded data from Dun and Bradstreet, Inc.	Convenience stores, grocery stores, supermarkets, fast-food and sit-down restaurants	Grocery stores positive (p < 0.05); convenience stores; restaurants; supermarket null
Cooksey-Stowers et al. (2017) USA	IV	Adults, nationwide database; general population	Access to food outlets	1 year	BMI	Obtained from USDA, self-reported survey	Ratio of fast-food restaurants and convenience stores to grocery stores and supermarkets; low access deﬁned as more than 1/10 mile from a supermarket store in an urban/rural area	Fast food restaurants, convenience stores, grocery stores, supermarkets	Positive (p < 0.05);
Zeng et al. (2019b) USA	IV	89 612 school age children; general population	Availability of convenience stores	1 year data	BMI	BMI health screenings from trained personnel	Count of convenience stores within 0.5 and 2 miles; data from geocoded lists purchased from Dun and Bradstreet, Inc.	Convenience stores	Positive (p < 0.05);
Anderson and Matsa (2011) USA	IV	13 470 individual adults' data; participants in rural areas	Proximity to restaurants	15 years	BMI	Behavioural Risk Factor Surveillance System (telephone survey)	Count of restaurant for every zip code in US, data from US Census ZIP Code Business Patterns	Full-service and limited-service restaurants	Null- participants in rural areas
Courtemanche and Carden (2011) USA	IV	1 644 094 individual adults' data	Availability of Walmart stores per 100 000 residents in a county	9 years	BMI	Behavioural Risk Factor Surveillance System (telephone survey)	Data on population from US Census Bureau, Walmart location from Holmes (2008)	Walmart stores	Positive (p < 0.05);

Note: Positive results imply that the environment exposure significantly increases dietary intake/purchases and/or BMI. Negative results imply that the environment exposure significantly decreases dietary intake/purchases and/or BMI. Lastly, null results imply that the environment exposure did not have any significant impact on dietary intake and/or BMI. IV stands for Instrumental Variable method, DID for Difference-in-Difference method, RCT -Randomised Controlled Trial, ITS for Interrupted Times Series. FV stands for fruit and vegetables. F stands for fruit; V stands for vegetable.

a Products were classified as healthy according to the Heart Foundation Tick nutrient profiling criteria.

b Meals meet nutritional criteria if they are ≤ 600 calories, ≤ 35% of total calories from fat, ≤ 10% saturated fat, ≤ 0.5 g of trans-fat, ≤ 640 mg of sodium.

c Fresh, frozen FV, low-fat dairy, whole grains, legumes, seeds, nuts and selected oils.

d Meals had to meet the requirements of the National Restaurant Association's Kids LiveWell program.

e One region is defined as having at least one large supermarket.

f Total fat, saturated fat, trans fat, FV, dark green and bright yellow vegetables.

g Burger King, Subway, KFC Wendy's, and Dunkin Donuts.

h Intake of solid fats, alcoholic beverages and added sugars.

i Daily caloric intake, daily carbohydrate intake, daily protein intake, and daily fat intake.

### 3.2 Study characteristics

From the 58 studies that met inclusion criteria, 57 were published after 2010, with an increasing trend over time in the use of experimental methods. 30 (51%) papers were randomized control trials (RCTs), and 28 quasi-experimental studies (14 difference-in-differences (DID), one interrupted-time-series (ITS), 11 instrumental variables (IV)) Definitions of the quasi-experimental methods are provided in Appendix 1. 55 studies were conducted in high income countries, with only two in upper middle-income countries. 41 (70%) studies investigated the relationship between food environment and health outcomes by focusing on the consumer food environment and 17 on the neighbourhood food environment (with one study examining both dimensions) (Jilcott Pitts et al., 2018a). 73% of the papers were on adults and 27% on children. 24 (35) % studies assessed obesity, measured by BMI, 44 (64%) assessed dietary intake (n = 13 FV, n = 31 general products), measured either objectively (e.g., measured consumption in a lab), or with surveys or purchases. RCTs were shorter with most lasting less than three months, while non-RCTs were longer than one year. In 26 of the studies participants were unaware they were part of a study. Five studies were in an experimental lab setting, including two that utilized a 3D virtual shop replicating real life shopping experience. Description of each study is provided in Table2.

### 3.3 Consumer food environment

From the 41 studies focusing on the consumer food environment, 17 examined the effect of in-kind (i.e., non-monetary) and financial incentives on diet and/or obesity, and 15 of saliency of healthy food and information, and nine on healthy food availability.

#### 3.3.1 In-kind and financial incentives

The effect of in-kind incentives on children was investigated by two studies reporting toy premiums significantly increased children’ purchases (tracked by scan cards) of healthier and low-calorie meals in fast-food restaurants, and FV snacks in local zoos(Hobin et al., 2012; Karpyn et al., 2017). Fifteen studies focused on the effect of financial incentives on dietary intake (Jilcott Pitts et al., 2018a; Geliebter et al., 2013; Waterlander et al., 2013a; Harnack et al., 2016; Ball et al., 2015; Kral et al., 2016; Bernales-Korins et al., 2017; Smith-Drelich, 2016; Franckle et al., 2018), purchases (Waterlander et al., 2013a, 2013b; Ball et al., 2015; Kral et al., 2016; Bernales-Korins et al., 2017; Smith-Drelich, 2016; Franckle et al., 2018; Blakely et al., 2011; Guan et al., 2018; Banerjee and Nayak, 2018; Polacsek et al., 2018; Gopalan et al., 2019), and BMI (Jilcott Pitts et al., 2018a; Geliebter et al., 2013; Waterlander et al., 2013a; Harnack et al., 2016; Kral et al., 2016), among adult samples. The type of financial incentives varied, including different levels of price reductions (Jilcott Pitts et al., 2018a; Geliebter et al., 2013; Waterlander et al., 2013a, 2013b; Harnack et al., 2016; Ball et al., 2015; Bernales-Korins et al., 2017; Blakely et al., 2011; Polacsek et al., 2018), coupons for targeted products (Franckle et al., 2018; Guan et al., 2018; Banerjee and Nayak, 2018), and cash back payments (reimbursements) (Kral et al., 2016; Smith-Drelich, 2016; Gopalan et al., 2019) based on purchasing behaviour.

Nine studies reported on the positive effect of price reductions (10%–50%) for healthy items in supermarkets on purchases (tracked by scan cards) and self-reported intake of FV and other healthy products, with effect size increasing on the discount (Jilcott Pitts et al., 2018a; Geliebter et al., 2013; Waterlander et al., 2013a, 2013b; Ball et al., 2015; Bernales-Korins et al., 2017; Blakely et al., 2011). Only one study reported null results on the effect of price reductions on self-reported FV intake and purchases (Jilcott Pitts et al., 2018a). This study is high risk of bias while the other eight are low or medium risk of bias. Three studies used financial incentives in the form of coupons to increase healthy food purchases (measured with in-store cards and transaction data) and self-reported FV and sugar-sweetened beverages (SSB) intake and found significant effects (i.e., increased FV intake and purchases) (Franckle et al., 2018; Guan et al., 2018; Banerjee and Nayak, 2018). These studies are low risk of bias.

Mixed results were found for the impact of cash back payments on healthy food purchases (measured by checking receipts) as one study incentivizing vegetable purchases in supermarkets found positive results (Smith-Drelich, 2016), while two studies that incentivized a variety of healthy items reported null (Kral et al., 2016; Gopalan et al., 2019). These studies are RCTs with low risk of bias score.

The effect of financial incentives (price discounts) on unincentivized products (unhealthy products) purchases was assessed by one (with low risk of bias score) study reporting null results (Waterlander et al., 2013b). One study (medium risk of bias score) combined 30% discount on FV with restriction on unhealthy products (candies, sweets baked goods, SSBs) and found significant results such that FV intake increased (p < 0.01) and energy from discretionary calories decreased (p < 0.01) as measured by self-reported dietary recalls among a low-income sample from USA. (Harnack et al., 2016)

None of the studied reviewed reported on financial incentives being effective in reducing self-reported consumption and purchases (measured with in-store cards) of sugar-sweetened beverages (SSBs) except when in combination with traffic light labelling on beverages products (Jilcott Pitts et al., 2018a; Geliebter et al., 2013; Ball et al., 2015; Franckle et al., 2018). The study that found significant results is low risk of bias (Franckle et al., 2018), whereas the others are medium and high risk of bias.

Further, five studies examined the effect of financial incentives on BMI and reported null results regardless of measurement method, self-reported (Jilcott Pitts et al., 2018a; Waterlander et al., 2013a), or measured by trained personnel (Geliebter et al., 2013; Kral et al., 2016). These studies varied in risk of bias with two being medium risk, one high risk, and two low risk of bias.

#### 3.3.2 Food saliency and information

Healthy food saliency (increasing visibility and reachability of healthier options) alone or in combination with information (about which foods are healthy) in supermarkets and restaurants significantly increased FVs intake and healthy food purchases for both children and adults(Anzman-Frasca et al., 2018; Knowles et al., 2019; Milliron et al., 2012).

Nine studies focusing on the effect of information (menu calorie labelling (Cantor et al., 2015; Vadiveloo et al., 2011; Marty et al., 2020; Petimar et al., 2019a; Petimar et al., 2019b; Finkelstein et al., 2011), FOP – traffic light (Hammond et al., 2013), numeric (Gustafson and Prate, 2019), warning signs (Grummon et al., 2019), labelling alone on the purchases of healthier products reported mainly null results). The exception being when information was conveyed through a warning sign (Grummon et al., 2019), or a FOP label tailored for specific populations (Gustafson and Prate, 2019), and health primes (recipe flyer featuring health and diet-related words) (Papies et al., 2014). Health primes decreased the purchases of energy-dense snacks for overweight adults (p < 0.01). (Papies et al., 2014).

One study, with a 15–20-month follow-up, assessed the impact of food saliency and information in local stores on adults BMI and found a negative effect (p < 0.01) (Gittelsohn et al., 2013). None of the studies examined the effect of information alone or in combination with health primes on BMI.

Most studies were low-risk of bias, except for two being high risk and one medium risk of bias (Gustafson and Prate, 2019; Grummon et al., 2019; Kristal et al., 1997). The low-risk of bias studies were with strong research designs (both experimental and quasi-experimental), good sample sizes (ranging from 56 to 7699), reasonable intervention durations of two to 14 months, and objectively measuring outcome with dietary intake data elicited via plate waste measurements in a laboratory, analysing shoppers’ baskets and BMI data measured by trained data collectors (Anzman-Frasca et al., 2018; Knowles et al., 2019; Milliron et al., 2012; Marty et al., 2020; Hammond et al., 2013; Papies et al., 2014; Gittelsohn et al., 2013). In five of these studies participants were unaware they were part of an experiment (Cantor et al., 2015; Vadiveloo et al., 2011; Petimar et al., 2019a, 2019b; Finkelstein et al., 2011).

#### 3.3.3 Accessibility/availability

The effect of increasing healthy food accessibility/availability in-stores (grocery, corner stores, supermarkets) on dietary intake and healthy food purchasing was investigated by nine studies reporting positive results for low-income children and null for low-income adults (Trude et al., 2018; Gittelsohn et al., 2017; Lent et al., 2014; Elbel et al., 2015, 2017; Dubowitz et al., 2015; Jilcott Pitts et al., 2018b; Laska et al., 2019; Cummins et al., 2014).

The studies on children are low risk of bias (Trude et al., 2018; Gittelsohn et al., 2017; Lent et al., 2014), while most of the ones on adults are high risk (Elbel et al., 2015, 2017; Dubowitz et al., 2015; Jilcott Pitts et al., 2018b; Laska et al., 2019; Cummins et al., 2014). Specifically, the studies on adults tested the impact of introducing a new supermarket with increased availability of healthy products in low-income neighbourhood, while the studies on children tested multi-level, multi-component interventions in local stores aimed at increased the purchases of healthy items.

BMI was not significantly affected by increasing the availability/accessibility of healthy products in studies on children and adults despite having long enough follow-ups of one to two years and regardless of BMI measurement type (Lent et al., 2014; Jilcott Pitts et al., 2018b; Cummins et al., 2014).

### 3.4 Neighbourhood food environment

Fifteen studies examined the neighbourhood food environment in the context of residential addresses and two were around schools. We did not identify any studies with suitable causal inference methodologies that focus on the neighbourhood food environment around workplaces.

Environment exposure was measured by counting the number of food outlets within different distances from participants’ home addresses. Distances varied from 0.5 miles to 1-mile in urban areas and from 2-miles to 10-miles for rural areas. Most of the studies focused either on the healthy food outlets or the unhealthy, with four studies examining both dimensions (Kapinos et al., 2014; Rummo et al., 2017; Cooksey-Stowers et al., 2017; Wang and Shi, 2012).

#### 3.4.1 Healthy food outlets

The effect of healthy food outlets on residents' dietary intake and BMI was examined by seven studies with differing methodologies and environment exposure (Jilcott Pitts et al., 2018a; Kapinos et al., 2014; Wang and Shi, 2012; Leone et al., 2018; Zhao et al., 2014; Zeng et al., 2019a; Olsho et al., 2015). One study reported that the number of supermarkets within 5 km (Murray et al., 2020) of children's home address did not have a significant effect on their nutrient intake (Wang and Shi, 2012).

The effect of healthy food outlets on FV intake among adults from low-income neighbourhoods was examined by three studies, with two studies reporting null results and the third one significant (Jilcott Pitts et al., 2018a; Leone et al., 2018; Olsho et al., 2015). The former two found no effect on self-reported FV intake after the introduction of a weekly veggie van in the neighbourhood (Leone et al., 2018; Olsho et al., 2015). In the former two studies, however, low exposure to the intervention was reported. In one of the studies only 21% of the respondents in the neighbourhood where the veggie van was introduced reported knowledge of it and only 8% reported shopping there (Olsho et al., 2015). The study that found significant results analysed the effect of distance to individual's primary grocery store from their home addresses on their self-reported FV intake and found significant and negative results (p < 0.05) (Jilcott Pitts et al., 2018a). The two studies that found null results are low risk of bias while the study that found significant is high risk of bias as it relied on self-reported data, had high attrition rate and did not account for important confounders.

Studies examining whether availability and distance to healthy food outlets influences residents’ BMI reported significant and negative results for low-income children and college dormitory students (BMI measured by trained personnel) such that the availability of grocery store within ¼ mile of residency and the opening of a new supermarket within 1 mile decreases BMI (Kapinos et al., 2014; Zeng et al., 2019a). While, the density of grocery stores within low-income families residencies was found to not have a significant effect on their self-reported BMI (Glanz et al., 2005; Zhao et al., 2014; Zeng et al., 2019a). These three studies utilized very different study designs, with one being DID, and two RCT but all being strong studies scoring low risk of bias.

#### 3.4.2 Unhealthy food outlet

The effects of unhealthy food outlets around a participant's home on fast-food consumption and BMI was examined by 12 studies which reported varied results based on the individuals age, area of living and ethnicity (Kapinos et al., 2014; Rummo et al., 2017; Cooksey-Stowers et al., 2017; Wang and Shi, 2012; Asirvatham et al., 2019; Zeng et al., 2019b; Dunn et al., 2012; Anderson and Matsa, 2011; Chen et al., 2013; Courtemanche and Carden, 2011; Alviola et al., 2014; Qian et al., 2017; Dunn, 2010).

Among a sample of children in China it was found that each additional wet market within 5 km (Murray et al., 2020) was associated with an increased self-reported daily caloric intake (p < 0.01), protein intake (p < 0.01) and fat intake (p < 0.01) (Wang and Shi, 2012). No effects were found for the number of fast-food restaurants (Wang and Shi, 2012).

Studies examining children’ BMI (measured by trained personnel) reported that one mile decrease in distance to fast food restaurants and convenience stores around schools and residential home addresses significantly increased BMI scores (p < 0.01), regardless of children SES but stronger effects were observed for girls when compared to boys (Asirvatham et al., 2019; Zeng et al., 2019b; Alviola et al., 2014; Qian et al., 2017).

Among adult samples all evidence comes from the US. In rural areas, proximity to fast food restaurants and the number of fast-food outlets within one and three miles were associated with increased self-reported fast-food consumption for non-whites (Black and Hispanic), but not for white adults (Dunn et al., 2012; Anderson and Matsa, 2011). Another study with a predominantly white sample found that while distance to fast-food restaurants increased the frequency of going to a fast-food restaurant the increased caloric intake was only marginally higher than when eating at home (35 calories more per day) (Chen et al., 2013). While a national US study found that food-swamps were associated with obesity rates (Cooksey-Stowers et al., 2017), evidence from individual level data showed that proximity to fast-food restaurants and density of fast-food restaurants was only associated with increased BMI in least populated areas (Courtemanche and Carden, 2011), and areas with low and middle population density (Dunn et al., 2012; Chen et al., 2013; Dunn, 2010), with most effects observed only for females (Courtemanche and Carden, 2011; Dunn, 2010), and Black and Hispanic (Dunn et al., 2012; Dunn, 2010). Null effects were reported for white adults (Kapinos et al., 2014; Rummo et al., 2017; Dunn et al., 2012; Anderson and Matsa, 2011; Dunn, 2010).

All studies are low risk of bias as they employed strong designs, with long durations, and participants were unaware they were part of an experiment.

## 4 Discussion

### 4.1 Summary of findings

We synthesized and appraised findings from quantitative studies that deploy methodologies suitable for causal inference to assess the causal impact of the consumer and neighbourhood food environment on dietary intake and obesity. Studies that assessed dietary intake focused on a range of outcomes such as FV or general nutrient, food intake and purchases, while obesity outcomes were measured only by BMI. The studies included a broad range of interventions and exposures to the consumer and the neighbourhood food environments. Most studies were of high quality, and the significant relationships between diet or obesity outcomes were mostly influenced by the type of intervention and exposure measured.

### 4.2 Interpretations and implications

While there have been systematic reviews in this area of research, we build on the evidence base by considering the broader exposure to obesogenic food environments, and assessing their causal impact on dietary intake, quality, and obesity related outcomes.

For the consumer food environment our results confirm and complement previous findings on financial incentives by suggesting that price discounts and coupons could significantly increase the intake and purchases of healthy foods among children and adults from different socioeconomic backgrounds (Cameron et al., 2016; An, 2013). The exception is for cash back payments which null effects on outcomes could be due to research design limitations such as temporal separation between the shopping and the cash back payments (Kral et al., 2016; Smith-Drelich, 2016; Gopalan et al., 2019). The added effort of going to the research centre, showing the receipts, and waiting for payment might diminish the effect of the financial incentive, especially for the targeted samples that were high income (Kral et al., 2016; Smith-Drelich, 2016; Gopalan et al., 2019).

Further, price discounts and coupons were effective in increasing the purchases and self-reported consumption of healthy products, but there was little evidence on their effect on other unincentivized products (Harnack et al., 2016). More evidence is needed to determine how financial incentives affect overall diets as discounts may not necessarily stop shoppers from buying unhealthy non-discounted products (Harnack et al., 2016). The lack of systematic assessment of the impact of incentives on non-incentivized foods together with no effect on SSBs could be a reason for the nonsignificant results on BMI (Jilcott Pitts et al., 2018a; Geliebter et al., 2013; Waterlander et al., 2013a; Harnack et al., 2016; Kral et al., 2016). Other potential reasons include: the selective implementation of the interventions in one or few supermarkets, not covering the broad range of places people source food from; interventions targeting a narrow set of foods that do not represent a significant part of individuals diets; short-lived incentives (ranging from eight weeks to six months) (Jilcott Pitts et al., 2018a; Geliebter et al., 2013; Waterlander et al., 2013a; Harnack et al., 2016; Kral et al., 2016). With regards to the latter, Waterlander and colleagues indicated that once the discount was removed all effects disappeared (Waterlander et al., 2013a). Studies that reported significant effects on BMI were at least one year long, suggesting that longer interventions and follows up are needed to observe significant changes in BMI. This is consistent with previous findings where 12-month interventions (in comparison to 3-month interventions) were observed to have greater and sustained effects on BMI (Ahern et al., 2017).

Increasing healthy food saliency in supermarkets and restaurants significantly increased healthy food consumption and reduced BMI (Anzman-Frasca et al., 2018; Knowles et al., 2019; Milliron et al., 2012; Marty et al., 2020; Gittelsohn et al., 2013). Health primes significantly decreased the purchases of energy dense foods among overweight adult populations (Papies et al., 2014). Also, health warning signs on SSBs and tailored FOP labelling on healthy products significantly increased healthy food purchases (Gustafson and Prate, 2019; Grummon et al., 2019). However, only providing information in the form of calorie and traffic light labels on products and menus did not significantly affect customers purchasing behaviour which is in line with previous evidence (Bleich et al., 2017; Cantor et al., 2015; Vadiveloo et al., 2011; Petimar et al., 2019a, 2019b; Finkelstein et al., 2011).

Also, increasing the accessibility/availability of healthy foods in stores alone did not significantly affect dietary intake and BMI, especially for adult populations and more research is required for children (Gittelsohn et al., 2013; Trude et al., 2018; Lent et al., 2014; Elbel et al., 2015, 2017; Dubowitz et al., 2015; Jilcott Pitts et al., 2018b; Laska et al., 2019; Cummins et al., 2014). Most of the studies focused on increasing the accessibility/availability of healthy foods by opening a new supermarket in the neighbourhood (Elbel et al., 2015, 2017; Dubowitz et al., 2015; Jilcott Pitts et al., 2018b; Laska et al., 2019; Cummins et al., 2014), not controlling for potential spill over effects on nearby control supermarkets. Also, it could be that individuals continued shopping in the stores that they used to go before the new supermarket opened, not adopting the new supermarket as their primary grocery store. This has been shown in one study as after a one-year follow-up few residents correctly identified the new supermarket (Elbel et al., 2015). Moreover, null results could be due to the effect not being strong enough for the targeted low-income sample, since discounted FV are often more expensive than processed food and spoil faster (Dubowitz et al., 2015).

Therefore, consistent with previous evidence, multifaced interventions that require little effort from the targeted groups (i.e., low agency) could be more conducive of improved healthy behavioural changes (Cameron et al., 2016; Adams et al., 2016).

In terms of the *neighbourhood food environment*, the availability of- or distance to-healthy food outlets did not affect the dietary intake and BMI of adults, while significant results were reported among children and college students (Kapinos et al., 2014; Zeng et al., 2019b). However, one of the quasi-experimental studies that found significant results on children only considered two pre-intervention periods not enabling testing for parallel trends nor residential sorting (Zeng et al., 2019a). Further, the study did not consider the effect of healthy food outlets such as grocery stores and farmer's markets. These concerns were mitigated in the RCT study that reported null results (Leone et al., 2018). The null effects among adult samples could be due to low intervention exposure and although participants reported shopping there, the purchasing data indicated otherwise, highlighting low compliance, and suggesting individuals kept buying in their preferred outlets (Leone et al., 2018; Olsho et al., 2015).

Further, the exposure to unhealthy food environments appears detrimental for children as the availability and proximity of fast-food restaurants and convenience stores around both homes and schools was associated with significantly increased BMIs, regardless of children SES (Asirvatham et al., 2019; Zeng et al., 2019b; Alviola et al., 2014; Qian et al., 2017). Only one study reported that the density of food outlets 5 km within children's homes was not significantly associated with BMI (Wang and Shi, 2012). However, in this study the density of food outlets was measured as the number of outlets without considering their size biasing actual food access, as food retailers tend to consolidate (Wood, 2013).

Among adults' unhealthy food environments were associated with increased resident's BMI and worsened dietary intake in women, black and Hispanics in low densely populated environments (Rummo et al., 2017; Cooksey-Stowers et al., 2017; Dunn et al., 2012; Anderson and Matsa, 2011; Chen et al., 2013; Courtemanche and Carden, 2011; Dunn, 2010). Most studies did not factor healthy food availability what may explain differences between high and low population density settings. Previous reviews found limited evidence for the relation between neighbourhood food environment and health-related outcomes, suggesting that null results could be due to failure to use a causal framework and failure to control for neighbourhood self-selection (Gamba et al., 2015; Feng et al., 2010; Williams et al., 2014; Casey et al., 2014). We complement this literature and provide a more granular level on these associations.

### 4.3 Strengths and limitations

Previous reviews report that the most common study limitations were failure to use a causal framework what might explain the predominant null results (Gamba et al., 2015; Feng et al., 2010; Williams et al., 2014; Casey et al., 2014). Therefore, a strength of this review is that we included only studies that use causal inference methodologies. This allows us to critically appraise studies that account for neighbourhood self-selection bias, endogeneity issues, and unobservable confounders. Other strengths include the holistic overview of the effect of the built food environment considering both the consumer and neighbourhood food environments on dietary and anthropometric related outcomes. We included evidence from any type of food outlet, did not limit to any specific interventions nor elements of the food environment, nor to any geographic region and included evidence for adults as well as for children. Although we found only two studies examining the food environment around schools and none on the food environment around workplaces, we did search for this evidence, what is another strength and novelty of this review as previous literature has been limited to residential addresses.

Despite these strengths, this review has several limitations. A limitation of our study is the lack of a research protocol. While we informally discussed the research design, scope, and methods of our review before conducting the research and adhered to the plan, the use of a protocol would have provided an opportunity to clarify definitions operationalised in the study from the outset, such as the definition of physical and virtual food environments.

Although we focused on papers with causal inference methods, these focused on certain elements of the food environment not accounting for the availability of certain foods at home, workplaces, and schools. This is due to the aggregated nature of data in most contributions that do not map holistically the different environments individuals are exposed to during their daily activities. This could explain the weak effects found on BMI. While there are a lot of cross-sectional or qualitative studies on the topic, we found only two studies with causal inference methodologies that evaluate the effect of food environments around schools on diet and obesity and we did not find any that examine the environment around workplaces. Therefore, a future area of research is the assessment of the role of food environments considering the full heterogeneity of environments to which individuals are exposed, leveraging data that maps throughout the day how individuals access food using travel patterns rather than merely home addresses. Also, a potential issue related to generalizability of these results could be that even though research was not limited to a specific geographic region, most of the studies were from western countries, specifically from the USA. Future research ought to investigate the relationship between the food environment and obesity and dietary intake in other countries.

Limitations of the revised articles include inaccurate datasets to identify food sources, categorizations of food sources based on generalized types, or the inclusion of a limited range of food sources. Furthermore, methodological choices matter when defining environmental exposure or access to food sources. For instance, a common strategy used to define exposure is to use administrative areas such as block groups, census tracks, or zip codes. However, this could be problematic as there might be highly uneven exposures within administrative boundaries.

## 5 Conclusion

Overall, the results suggest that elements of the environment impact diets and obesity differently. To the extent to which exposure to those elements varies across income, sex, age, and ethnicity, one size fits all interventions will not suffice to promote healthier diets and reduce ubiquitous health inequalities in nutritional related outcomes. Importantly our findings suggest that low agency interventions (e.g. financial incentives at the point of purchase, health primes, accessibility/availability of healthy/unhealthy food) tend to be more effective in improving diets and mitigating obesity than high agency interventions (e.g. informational interventions such as front of pack labelling, or financial incentives that require effort from consumers such as cash back incentives) (Adams et al., 2016). Therefore, while interventions in the built food environments have the potential to improve nutritional intake and public health through their role in affecting obesity/dietary outcomes, low agency interventions and those that are personalized to account for different levels of exposure to the elements of the built environment are more likely to be effective.

## Role of the funding source

The funder had no role in study design, data collection, analysis, interpretation, or writing of the report.

## 6 Contributions

MM and FS were responsible for the conceptual design of the review. PA conducted the research. MM, PA, EP, FS screened the papers and devised themes. All authors contributed to the drafting of the paper.

## Declaration of interest

GF declare grants from Nestle, grants from Heptares, other from Melico Science, outside the submitted work. FS, MM, EP, DK, and PA have nothing to disclose.

## Data sharing

Extracted data are available upon request to the corresponding author.

## Footnotes

Supplementary data to this article can be found online at https://doi.org/10.1016/j.socscimed.2022.114879.
