# Supplementary material for: The impact of the consumer and neighbourhood food environment on dietary intake and obesity-related outcomes: A systematic review of causal impact studies
Source: Soc Sci Med. 2022 Apr;299:114879. doi: 10.1016/j.socscimed.2022.114879 (PMC8987734; doi:10.1016/j.socscimed.2022.114879)
Supplement: Multimedia component 2 [file mmc2.docx]

# **SUPPLEMENTARY APPENDIX**

Manuscript title: The impact of the consumer and neighbourhood food environment on dietary intake and obesity-related outcomes: A systematic review of causal impact studies.

**Appendix 1. PICO table**

| **PICO feature** | **Criteria** |
| --- | --- |
| **Population** | Adults and children of any sex, ethnicity, SES and country of origin.  **Excluded** **populations**: adults or children with a critical illness or severe co‐morbidities or special populations (e.g. blind, physically disabled) or studies with very targeted populations (e.g. athletes, religious groups, pregnant women, etc). |
| **Intervention(s)** | Objective (e.g. [GIS] mapping or neighbourhood audits) or perceived (e.g. self- report) elements of the built food environment or interventions in the built environment that could affect dietary behaviour and obesity. We examine the built food environment which encompasses the neighbourhood and consumer food environment:  **Neighbourhood food environment** – area around one’s household, school, workplace – e.g. proximity, density, or diversity of food available near residents’ home addresses, school, or/and workplace.  **Consumer food environment** – food environment within any food outlets – e.g., types of food available, promotions, placements, range of choice, and nutrition information.  **Excluded interventions**: all studies that did not examine the neighbourhood or consumer food environment. Studies focusing on the organisational food environment (the food environment inside homes, schools, workplaces); and studies focusing on the online food environment. |
| **Comparison(s)** | Individuals not exposed to the food environment element/intervention being assessed. |
| **Outcomes** | Objective or self-reported measurement of health-related behaviours in terms of dietary intake and obesity.  **Dietary outcome** – fruit intake/purchases, vegetable intake/purchases, energy-dense food intake/purchases, fast foods, sugary drinks intake/purchases, dietary quality indices, nutrient intake/purchases, calories intake/purchases, healthy products intake/purchases.  **Obesity outcome -**BMI, waist circumference, weight  **Excluded outcomes***:* studies examining health outcomes related to tobacco consumption or alcohol consumption or physical activity, or any other that is not related to dietary intake and obesity |
| **Study design** | Any study of human subjects examining at least one built food environment metric and its association with at least one health related outcome (dietary intake or obesity). Any intervention that deploys quantitative methods suitable for causal inference including randomized control trials (RCT), and quasi-experimental designs such as interrupted time series (ITS), instrumental variable (IV), difference-in-difference (DID). The latter are very broadly defined statistical techniques used in econometrics and quantitative research in the social sciences that attempts to mimic an experimental research design using observational data.  **Excluded study designs** non-human samples, qualitative studies (e.g. focus groups, descriptive), observational studies without clear experimental or quasi-experimental designs, case studies and case reports, policy analysis, socioeconomic analysis. |

**Appendix 2. Search string and results from Ovid Medline. This search string was adapted for searches in Embase and PsychInfo (via Ovid), and EconLit (via EBSCO). The exact search string and results from these databases are available upon request to the corresponding author.**

| **[# ▲](https://ovidsp.dc1.ovid.com/sp-4.05.0b/ovidweb.cgi?&S=LAKNFPBLOIACCDACKPBKCHFOINBDAA00&Sort+Sets=descending)** | **Searches** |
| --- | --- |
| 1 | fruit* intake.mp. |
| 2 | fruit* consumption.mp. |
| 3 | vegetable* intake.mp. |
| 4 | vegetable* consumption.mp. |
| 5 | energy-dense food* consumption.mp. |
| 6 | eating behaviour.mp. |
| 7 | *Energy Intake/ |
| 8 | diet* habit*.mp. |
| 9 | energy dense food*.mp. |
| 10 | sugary drink*.mp. |
| 11 | sugar sweetened beverages.mp. |
| 12 | sugar sweetened drink*.mp. |
| 13 | Diet, Healthy/ |
| 14 | eat* habit*.mp. |
| 15 | eating index.mp. |
| 16 | food index.mp. |
| 17 | salt consumption.mp. |
| 18 | fat consump*.mp. |
| 19 | salt intake.mp. |
| 20 | fat intake.mp. |
| 21 | 1 or 2 or 3 or 4 or 5 or 6 or 7 or 8 or 9 or 10 or 11 or 12 or 13 or 14 or 15 or 16 or 17 or 18 or 19 or 20 |
| 22 | exp Obesity/ |
| 23 | obes$.mp. |
| 24 | ((bmi or body mass index) adj3 (gain* or increase*)).mp. |
| 25 | weight gain.mp. |
| 26 | (overweight or over weight or overeat$ or over eat$).mp. |
| 27 | Waist Circumference/ |
| 28 | Body Composition/ |
| 29 | Waist-Hip Ratio/ |
| 30 | 22 or 23 or 24 or 25 or 26 or 27 or 28 or 29 |
| 31 | 21 or 30 |
| 32 | (restaurant* or fast-food* or retail store* or convenience store* or supermarket* or chain grocer* or farmer* market* or shop*).mp. |
| 33 | (food adj3 (access or environment* or neighbourhood* or supply or supplies or desert* or store* or shop* or provide* or retail*)).mp. |
| 34 | Restaurants/ |
| 35 | nutriti* environment*.mp. |
| 36 | street food.mp. |
| 37 | 32 or 33 or 34 or 35 or 36 |
| 38 | (geographic* or geospatial*).mp. |
| 39 | (geolocation* or locat*).mp. |
| 40 | spatial*.mp. |
| 41 | (proximit* or local* or buffer or densit* or distance* or variet*).mp. |
| 42 | (school* or home* or workplace* or work*).mp. [mp=title, abstract, original title, name of substance word, subject heading word, floating sub-heading word, keyword heading word, organism supplementary concept word, protocol supplementary concept word, rare disease supplementary concept word, unique identifier, synonyms] |
| 43 | Regression Analysis/ |
| 44 | statistic*.mp. |
| 45 | Data Analysis/ |
| 46 | Models, Econometric/ |
| 47 | 31 and 37 |
| 48 | 38 or 39 or 40 or 41 or 42 or 43 or 44 or 45 or 46 |
| 49 | 47 and 48 |
| 50 | limit 49 to humans |

**Appendix 3. Risk of bias assessment table**

| Author/year/ref | Selection Bias - patients randomly assigned/recruited (yes =1, no = 0) | Is attrition rate right accounted for? (yes =1, no = 0) | Was the intervention long enough?  (for dietary intake or purchases more than 1 month is 1, for BMI more than 6 months is 1; less than those 0) | Was the environmental exposure objectively measured?  (collected by researchers counts as objective; self-reported exposure or defined on block groups or zip codes counts as not objective) | Was the dietary intake and/or BMI objectively measured? (sales data, purchases tracked with cards, receipt, measurements by trained personnel count as objective; all self-reported data counts as not objective) | Were all important confounding factors considered?  (1 if accounts for age, gender, SES or more, 0 if only some of those e.g. only age and gender) | Are the results of the treatment effect well reported and is the analysis coherent? (yes =1, no = 0) | Are the estimates of the treatment effect precise? (yes =1, no = 0) | Is the study a natural field experiment (participants are not aware of participation)? (yes =1, no = 0) | Can the results be applied to the local population, or in your context? (yes =1, no = 0) | **Score** |
| --- | --- | --- | --- | --- | --- | --- | --- | --- | --- | --- | --- |
| Hobin (2012)^37^ | 1 | 1 | 1 | 1 | 1 | 1 | 1 | 1 | 0 | 1 | 9 |
| Karpyn (2017)^38^ | 1 | 1 | 0 | 1 | 0 | 1 | 1 | 1 | 0 | 1 | 7 |
| Geliebter (2013)^39^ | 1 | 0 | 0 | 1 | 1 | 1 | 1 | 1 | 0 | 0 | 6 |
| Waterlander (2013)^40^ | 1 | 1 | 1 | 1 | 0 | 1 | 1 | 1 | 0 | 0 | 7 |
| Ball (2015)^42^ | 1 | 0 | 1 | 1 | 1 | 1 | 1 | 1 | 0 | 0 | 7 |
| Blakely (2011)^47^ | 1 | 1 | 1 | 1 | 1 | 1 | 1 | 1 | 0 | 0 | 8 |
| Polacsek (2017)^51^ | 1 | 1 | 0 | 1 | 1 | 0 | 1 | 1 | 0 | 0 | 6 |
| Harnack (2016)^41^ | 1 | 1 | 0 | 1 | 0 | 1 | 1 | 1 | 0 | 0 | 6 |
| Jilcott Pitts, (2018)^36^ | 0 | 0 | 1 | 1 | 0 | 0 | 1 | 1 | 0 | 0 | 4 |
| Gopalan (2019)^52^ | 1 | 0 | 1 | 1 | 1 | 0 | 1 | 1 | 0 | 1 | 7 |
| Kral (2016)^43^ | 1 | 1 | 0 | 1 | 1 | 1 | 1 | 1 | 0 | 0 | 7 |
| Waterlander (2013)^48^ | 1 | 1 | 0 | 1 | 1 | 1 | 1 | 1 | 0 | 0 | 7 |
| Guan (2018)^49^ | 0 | 1 | 1 | 1 | 0 | 1 | 1 | 1 | 1 | 0 | 7 |
| Bernales-Korins (2017)^44^ | 1 | 1 | 1 | 1 | 1 | 1 | 1 | 1 | 0 | 0 | 8 |
| Smith-Drelich. (2016)^45^ | 1 | 1 | 0 | 1 | 1 | 1 | 1 | 1 | 0 | 1 | 8 |
| Franckle (2018)^46^ | 1 | 1 | 1 | 1 | 1 | 1 | 1 | 1 | 0 | 1 | 9 |
| Banerjee (2018)^50^ | 1 | 1 | 1 | 1 | 0 | 1 | 1 | 1 | 0 | 0 | 7 |
| Anzman-Frasca (2018)^53^ | 1 | 1 | 1 | 1 | 1 | 1 | 1 | 1 | 0 | 1 | 9 |
| Cantor (2015)^56^ | 1 | 0 | 1 | 1 | 1 | 1 | 1 | 1 | 1 | 1 | 9 |
| Vadiveloo (2011)^57^ | 1 | 0 | 1 | 1 | 1 | 1 | 1 | 1 | 1 | 1 | 9 |
| Knowles (2019) ^54^ | 1 | 1 | 1 | 1 | 1 | 1 | 1 | 1 | 0 | 1 | 9 |
| Marty (2020)^58^ | 1 | 1 | 1 | 1 | 1 | 1 | 1 | 1 | 0 | 1 | 9 |
| Gittelsohn (2013)^66^ | 1 | 0 | 1 | 1 | 1 | 1 | 1 | 1 | 0 | 0 | 7 |
| Milliron (2012)^55^ | 1 | 1 | 1 | 1 | 0 | 1 | 1 | 1 | 0 | 1 | 8 |
| Kristal (1997)^67^ | 1 | 1 | 1 | 0 | 0 | 0 | 1 | 1 | 0 | 0 | 5 |
| Petimar (2019)^60^ | 0 | 0 | 1 | 1 | 1 | 0 | 1 | 1 | 1 | 1 | 7 |
| Petimar(2019)^59^ | 0 | 0 | 1 | 1 | 1 | 0 | 1 | 1 | 1 | 1 | 7 |
| Finkelstein (2011)^61^ | 0 | 1 | 1 | 1 | 1 | 0 | 1 | 1 | 1 | 0 | 7 |
| Papies (2014)^65^ | 1 | 1 | 0 | 1 | 0 | 1 | 1 | 1 | 0 | 1 | 7 |
| Hammond (2013)^62^ | 1 | 1 | 0 | 1 | 1 | 1 | 1 | 1 | 0 | 1 | 8 |
| Gustafson (2019)^63^ | 1 | 1 | 0 | 1 | 0 | 1 | 1 | 1 | 0 | 0 | 6 |
| Grummon (2019)^64^ | 1 | 0 | 0 | 0 | 1 | 1 | 1 | 1 | 0 | 0 | 5 |
| Gittelsohn (2017)^69^ | 1 | 1 | 1 | 1 | 0 | 1 | 1 | 1 | 0 | 0 | 7 |
| Lent (2014)^70^ | 1 | 0 | 1 | 1 | 1 | 1 | 1 | 1 | 0 | 1 | 8 |
| Trude (2018)^68^ | 1 | 1 | 1 | 1 | 0 | 1 | 1 | 1 | 0 | 0 | 7 |
| Jilcott Pitts (2018)^74^ | 0 | 0 | 1 | 1 | 0 | 0 | 1 | 1 | 0 | 0 | 4 |
| Cummins (2014)^76^ | 0 | 1 | 1 | 1 | 0 | 1 | 1 | 1 | 1 | 0 | 7 |
| Dubowitz (2015)^73^ | 0 | 0 | 1 | 1 | 0 | 1 | 1 | 1 | 1 | 0 | 6 |
| Elbel (2015)^71^ | 0 | 0 | 1 | 1 | 0 | 1 | 1 | 1 | 1 | 0 | 6 |
| Elbel (2017)^72^ | 0 | 0 | 1 | 1 | 0 | 1 | 1 | 1 | 1 | 0 | 6 |
| Laska (2019)^75^ | 1 | 1 | 1 | 1 | 1 | 1 | 1 | 0 | 1 | 1 | 9 |
| Zeng (2019)^83^ | 0 | 1 | 1 | 0 | 1 | 1 | 1 | 1 | 1 | 1 | 8 |
| Zhao (2014)^82^ | 1 | 0 | 1 | 1 | 0 | 1 | 1 | 1 | 1 | 1 | 8 |
| Leone (2018)^81^ | 1 | 1 | 1 | 1 | 0 | 1 | 1 | 1 | 0 | 0 | 7 |
| Olsho (2015)^84^ | 0 | 1 | 1 | 1 | 0 | 1 | 1 | 1 | 1 | 0 | 7 |
| Kapinos (2014) ^77^ | 1 | 1 | 1 | 1 | 0 | 1 | 1 | 1 | 1 | 0 | 8 |
| Alviola (2014) ^91^ | 1 | 1 | 1 | 0 | 1 | 0 | 1 | 1 | 1 | 1 | 8 |
| Asirvatham (2019)^85^ | 1 | 1 | 1 | 0 | 1 | 0 | 1 | 1 | 1 | 1 | 8 |
| Wang (2012)^80^ | 1 | 1 | 1 | 1 | 0 | 1 | 1 | 1 | 1 | 0 | 8 |
| Qian (2017)^92^ | 1 | 1 | 1 | 1 | 1 | 0 | 1 | 1 | 1 | 0 | 8 |
| Chen (2013)^89^ | 1 | 0 | 1 | 1 | 0 | 1 | 1 | 1 | 1 | 1 | 8 |
| Dunn (2012)^87^ | 1 | 1 | 1 | 1 | 0 | 1 | 1 | 1 | 1 | 0 | 8 |
| Dunn et al. (2010)^93^ | 1 | 1 | 1 | 1 | 0 | 1 | 1 | 1 | 1 | 0 | 8 |
| Rummo (2017) ^78^ | 1 | 0 | 1 | 0 | 1 | 1 | 1 | 1 | 1 | 1 | 8 |
| Cooksey-Stowers (2017)^79^ | 1 | 0 | 1 | 1 | 0 | 1 | 1 | 1 | 1 | 0 | 7 |
| Zeng (2019)^86^ | 1 | 0 | 1 | 0 | 1 | 0 | 1 | 1 | 1 | 1 | 7 |
| Anderson (2011)^88^ | 1 | 1 | 1 | 0 | 0 | 0 | 1 | 1 | 1 | 1 | 7 |
| Courtemanche (2011)^90^ | 1 | 1 | 1 | 1 | 0 | 0 | 1 | 1 | 1 | 1 | 8 |

Note: Quality concerns included: selection bias, not accounting for high attrition rate, too short follow up periods, not objectively measuring outcomes (obesity and dietary intake) and using self-reported data, not objectively measuring exposure, and using information from commercial databases, not accounting for all relevant confounding factors, and not being generalizable. Given the nature of the research assessed we also considered whether studies were natural field experiments in which participants are unaware they are part of a study or field experiments where, given the nature of the interventions assessed, participants are not blind to the treatment. The scoring tool consisted of 10 criteria, with scores 1 or 0. For instance, if the answer to the question “Was the dietary intake and/or BMI objectively measured?” is yes, then 1 point is given, 0 points are given otherwise. A final quality concern score was calculated by summing the points for each study. The highest score was 10 and studies were considered high risk if they had a score equal or lower than 5, medium risk if they had a score of 6, and low risk if they had a score equal or bigger than 7*.*
